# Real-World Mobile Health Implementation and Patient Safety: Multicenter Qualitative Study

**DOI:** 10.2196/71086

**Published:** 2025-04-29

**Authors:** Jing Jing Su, Michelle Hui Shan Chan, Gabriela Lima de Melo Ghisi, Rick Yiu Cho Kwan, Arkers Kwan Ching Wong, Rose Lin, Jerry Wing Fai Yeung, Qijun He, Garyfallia Pepera, Ladislav Batalik

**Affiliations:** 1 School of Nursing Tung Wah College Hong Kong China (Hong Kong); 2 Translational Research Centre for Digital Mental Health Tung Wah College Hong Kong China (Hong Kong); 3 Community Nursing Service Heaven of Hope Hospital Hong Kong China (Hong Kong); 4 KITE Research Institute University Health Network Toronto, ON Canada; 5 Department of Physical Therapy Temerty Faculty of Medicine University of Toronto Toronto, ON Canada; 6 School of Nursing The Hong Kong Polytechnic University Hong Kong China (Hong Kong); 7 Elaine Hubbard Center for Nursing Research on Aging School of Nursing University of Rochester Medical Center Rochester, NY United States; 8 School of Journalism and Communication Shanghai University Shanghai China; 9 Clinical Exercise Physiology and Rehabilitation Research Laboratory, Department of Physiotherapy Faculty of Health Sciences University of Thessaly Lamia Greece; 10 Department of Rehabilitation University Hospital Brno Brno Czech Republic; 11 Department of Public Health Faculty of Medicine Masaryk University Brno Czech Republic; 12 Department of Physiotherapy and Rehabilitation Faculty of Medicine Masaryk University Brno Czech Republic; 13 Rehabilitation Clinic Faculty of Medicine Masaryk University Brno Czech Republic

**Keywords:** mHealth, mobile health, patient safety, qualitative study, real-world implementation, mobile health technologies

## Abstract

**Background:**

Mobile health (mHealth) is increasingly being used in contemporary health care provision owing to its portability, accessibility, ability to facilitate communication, improved interprofessional collaboration, and benefits for health outcomes. However, there is limited discourse on patient safety in real-world mHealth implementation, especially as care settings extend beyond traditional center-based technology usage to home-based care.

**Objective:**

This study aimed to explore health care professionals’ perspectives on the safety aspects of mHealth integration in real-world service provision, focusing on Hong Kong Special Administrative Region (SAR) and Wuhan city in mainland China. In Hong Kong SAR, real-world mHealth care provision is largely managed by the Hospital Authority, which has released various mobile apps for home-based care, such as Stoma Care, Hip Fracture, and HA Go. In contrast, mHealth care provision in Wuhan is institutionally directed, with individual hospitals or departments using consultation apps, WeChat mini-programs, and the WeChat Official Accounts Platform (a subapp within the WeChat ecosystem).

**Methods:**

A multicenter qualitative study design was used. A total of 27 participants, including 22 nurses and 5 physicians, from 2 different health care systems were interviewed individually. Thematic analysis was used to analyze the data.

**Results:**

The mean age of the participants was 32.19 (SD 3.74) years, and the mean working experience was 8.04 (SD 4.05) years. Most participants were female (20/27, 74%). Nearly half of the participants had a bachelor’s degree (13/27, 48%), some had a master’s degree (9/27, 33%), and few had a diploma degree (3/27, 11%) or a doctoral degree (2/27, 7%). Four themes emerged from the data analysis. Considering the current uncertainties surrounding mHealth implementation, participants emphasized “liability” concerns when discussing patient safety. They emphasized the need for “change management,” which includes appropriate referral processes, adequate resources and funding, informed mHealth usage, and efficient working processes. They cautioned about the risks in providing mHealth information without ensuring understanding, appreciated the current regulations available, and identified additional regulations that should be considered to ensure information security.

**Conclusions:**

As health care systems increasingly adopt mHealth solutions globally to enhance both patient care and operational efficiency, it becomes crucial to understand the implications for patient safety in these new care models. Health care professionals recognized the importance of patient safety in making mHealth usage reliable and sustainable. The promotion of mHealth should be accompanied by the standardization of mHealth services with institutional, health care system, and policy-level support. This includes fostering mHealth acceptance among health care professionals to encourage appropriate referrals, accommodate changes, ensure patient comprehension, and proactively identify and address threats to information security.

## Introduction

Mobile health (mHealth) involves the use of mobile devices, such as advanced technologies, smart wearables, mobile phones, and patient monitoring tools, to support remote health care delivery [1,2]. mHealth is widely adopted worldwide, and it aims to improve health care accessibility [3], cost-effectiveness [4], and efficiency across diverse health care settings. Such platforms empower individuals to manage chronic illnesses and adopt healthier lifestyles (eg, exercise and smoking cessation), and promote self-diagnosis at their discretion [5]. Patients can access specialized consultations, purchase medications without additional travel, and monitor and interpret their health data in real-time through wearable devices [6,7]. Additionally, mHealth enhances communication among health care professionals, fosters interdisciplinary collaboration, and involves caregivers in the care process [8]. Beyond its technological capacity, mHealth redefines health care workflows and reallocates roles among professionals, patients, and their families [9,10].

While health care professionals adopt mHealth to empower patients’ active engagement in their own health care [10], they also encounter challenges related to patient safety [11]. Unlike traditional health care, where professionals collect and verify health information, mHealth often requires users to upload their own health data, which is then processed by digital platforms [12]. This shift introduces risks, including the absence of in-person assessments, limited nonverbal cues during online consultations [13], and disparities in digital literacy [14]. The proliferation of mHealth apps and devices, with a large volume of text, audio, or video content, also complicates the evaluation of content validity and reliability [7].

While existing research on mHealth safety has largely been conducted in academic settings or outside real-world care delivery systems and is known to cease with funding expiration [15,16], there is limited evidence on its integration into routine care provision. Literature on interventional studies has highlighted issues, such as content accuracy, lack of professional involvement [15], and failure to incorporate user feedback for enhancing usability and medical reliability [17]. Studies have also revealed specific concerns from such controlled settings, including data security [18], privacy, adverse events related to remote exercise promotion [19], and professional-client relationships [2]. However, as mHealth transitions from professionally guided disease management to patient-driven preventive care, where individuals take on greater responsibility for their own health, potential safety risks, such as exacerbation of health inequities and miscommunication [13,20], become significant [9,10]. This gap in understanding how mHealth is applied in real-world contexts and its associated safety challenges in routine care provision needs further exploration [10].

When studying this issue more deeply, it becomes clear that safe design and development, appropriate implementation, workflow integration, and responsibility statements are essential for enhancing patient safety [21]. According to the structure-process-outcome (SPO) framework by Donabedian [22], the use of health technologies can be assessed and enhanced through 3 components: structure (ie, mobile apps and personnel), process (ie, care provision content), and outcome (ie, health and costs) [9]. Ensuring safety when delivering care via technologies involves different aspects of these 3 categories and their interrelationships. Specifically, mHealth platforms should be designed and developed to support health goals and workflows, and organizations must configure health technologies when adopting available mHealth. Furthermore, with the remarkable increase in the volume of literature on safety issues surrounding mHealth, it is important to extend the understanding from research to real-world implementation. The intricacies of safety considerations are rooted in the nature of mHealth, attitudes toward change and invention, resource availability, compatibility with existing health care systems, and impact [23]. Additionally, similar mHealth interventions may be used across various organizations, which may be related to the mentality and characteristics of people and the operation of systems.

In China, the real-world implementation of mHealth varies significantly between Hong Kong Special Administrative Region (SAR) and mainland China due to the “one country two system” policy [24]. Hong Kong’s Hospital Authority administers mHealth services through a standardized suite of apps, supporting health care institutions financially to provide mHealth services [24]. Various physical, electronic, and management measures, such as setting up a data protection unit in the Hospital Authority office, regulating data collection and storage, denying unauthorized access, and providing access with informed consent, were adopted to secure personal data under the Personal Data (Privacy) Ordinance [25]. Health care professionals in community settings, especially community nurses, frequently use these tools during remote consultations or home visits. Conversely, in mainland China, mHealth adoption is hospital-centric, with public hospitals offering diverse platforms funded through their own resources to support remote consultation and home-based care [26]. The National Health Commission released a regulatory policy stating that mHealth services should be based within onsite health care institutions, should be provided by registered health care professionals, should be offered to patients diagnosed with common chronic illnesses, and should protect patient information. The regulation focuses on guiding diagnostic and pharmacological activities, but widespread mHealth usage for patient education, self-care empowerment, and lifestyle or psychosocial consultation is not covered [27]. Given these variations in implementation and the associated safety challenges, understanding health care professionals’ perspectives in these distinct contexts can provide valuable insights into optimizing mHealth integration in real-world settings. Thus, this study explores health care professionals’ perspectives on the safety aspects of mHealth integration in real-world service provision, with a focus on Hong Kong SAR and Wuhan city in mainland China.

## Methods

### Study Design

This study employed a multicenter qualitative descriptive design, which is well-suited for exploring human experiences and gaining insights into subjective perspectives on the safety aspects of mHealth usage [28]. A multicenter approach was chosen to capture diverse perspectives, enhance representation, and improve the transferability of findings [29]. The study presentation was guided by the 21-item Standards for Reporting Qualitative Research checklist [30].

### Setting, Participants, and Sampling

This study was conducted in Hong Kong SAR and Wuhan city in mainland China from May 2022 to August 2024. Both locations are characterized by high internet penetration rates and widespread mHealth usage [31,32]. Eligible participants were health care professionals who used mHealth for health service provision as initiated by their institutions, departments, or health governing authorities. Health care professionals working in nondirect care roles, such as technical support or appointment booking hotlines, were excluded. Since both settings lack formal training for staff in using mHealth and some mHealth follow-ups require 6 to 12 weeks, those with less than 3 months of experience in providing mHealth services were excluded to ensure a thorough understanding of the topic. For sample homogeneity, only health care professionals offering real-world mHealth care services in public health care institutions (eg, hospitals and community health care centers) were included.

Participants were recruited through purposive sampling, leveraging poster advertisements distributed via alumni groups, social media platforms, and word of mouth. This approach allowed the study researchers to intentionally select participants with substantial knowledge and experience in mHealth usage for in-depth interviews [33].

### Data Collection

Semistructured individual in-depth interviews were conducted to capture the participants’ perspectives while ensuring relevance to the study objective [34]. An interview guide was developed based on the authors’ experiences, the literature, and a framework (Multimedia Appendix 1). Two female authors (JJS and MHSC), both with extensive experience in mHealth service provision and qualitative interviewing, conducted the interviews. Their familiarity with the topic enabled a nuanced understanding of participants’ experiences, aligning with the notion that interviewer-participant dynamics exist on a continuum rather than an insider-outsider dichotomy [35]. Both interviewers received extensive training in conducting qualitative interviews to ensure neutrality. Additionally, a standard interview guide was used and a pilot interview was conducted, which was followed by a briefing, to ensure data integrity [36]. To maintain confidentiality, no identifying information, such as participants' names or institutional details, was collected [37]. Probing questions, neutral interview locations with privacy (eg, a quiet coffee shop or garden), and a nonjudgmental and respectful attitude were employed to minimize social desirability bias and encourage authentic responses [38].

Interviews lasted for 40 to 60 minutes, were audio-recorded, and were accompanied by field notes to capture nonverbal cues. Depending on participants’ preferences, interviews were conducted either in person or online (via videoconferencing) to accommodate their schedules. The use of 2 interview channels was supported by the literature, as both methods yield similar levels of self-disclosure and equivalent thematic content [39,40]. The sample size was determined based on the principle of data saturation, with 2 additional interviews conducted after no new findings emerged from the 25th interview to confirm saturation [41].

### Data Analysis and Trustworthiness

Thematic analysis was used, which can identify patterns within and across data regarding participants’ views, experiences, and practices [42]. The audio recordings were transcribed verbatim alongside field notes. Transcripts were reviewed for accuracy against the original audio recordings by interviewers. Two researchers (JJS and MHSC) independently read and reread the transcripts to immerse themselves into the data and made notes to identify the initial codes [36]. After independently coding 4 interviews, the researchers compared their codes for consistency and resolved discrepancies through discussion, updating the codebook accordingly. Subsequent transcripts were coded independently, with regular adjudication sessions held to refine the codebook. Data saturation was reached at the 16th interview with no themes emerging. However, the researchers completed 27 interviews to ensure a richly textured understanding of the topic [43]. Codes with similar meanings or conceptual relationships were grouped into subthemes and themes using constant comparative analysis. Final themes were determined through extensive team discussions. Dependability was ensured by maintaining an audit trail documenting the rationale for coding decisions and theme development. Member checking was conducted with 7 participants from Hong Kong (n=3) and Wuhan (n=4) to validate the findings.

### Ethical Considerations

Ethics approval was obtained from the Institutional Review Boards of Hong Kong Polytechnic University (HSEARS20240131005) and Shanghai University (ECSHU2022046). Participants were informed of their right to withdraw at any time without any consequences. Written informed consent was obtained from participants before interviews commenced.

## Results

### Sociodemographic Results

A total of 27 health care professionals were recruited and interviewed, including 20 professionals (5 physicians and 15 nurses) from hospitals in Wuhan and 7 nurses from community health care settings in Hong Kong SAR. The mean age of the participants was 32.19 (SD 3.74) years, with a mean working experience of 8.04 (SD 4.05) years. Most participants were female (20/27, 74%). Nearly half of the participants had a bachelor’s degree (13/27, 48%), some had a master’s degree (9/27, 33%), and few had a diploma degree (3/27, 11%) or a doctoral degree (2/27, 7%). Participants from Hong Kong SAR shared using various mobile apps published by the Hospital Authority for home-based care, such as Stoma Care, Hip Fracture, and HA Go. On the other hand, participants from Wuhan shared using consultation apps of their hospitals, WeChat mini-programs, and the WeChat Official Accounts Platform (a subapp within the WeChat ecosystem).

### Themes and Subthemes

**Figure 1:**
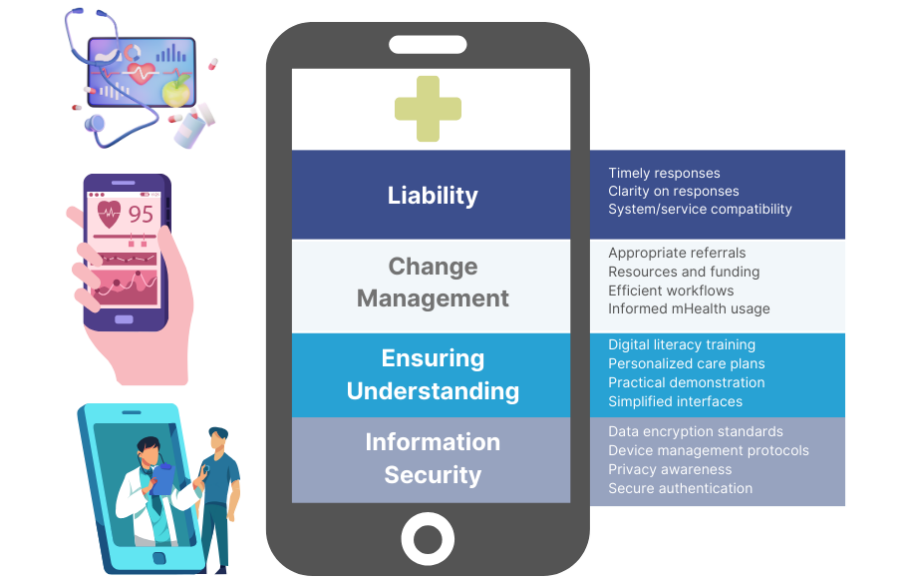
Key themes and subthemes addressing patient safety in mobile health (mHealth) implementation.

Four overarching themes and 4 subthemes emerged, specifically addressing the multifaceted dimensions of safety concerns, including the theoretical, technical, organizational, and patient-centered aspects of mHealth use. All 4 themes were covered by participants from 2 different health care systems, with some differences in subthemes further elaborated. Figure 1 illustrates the key themes and subthemes identified. 

### Theme 1: Liability

Liability emerged as a central concept for health care professionals when discussing patient safety in the context of mHealth usage. While participants used varied terms, such as “who,” “what if,” and “how,” their insights collectively illuminated the multifaceted risks tied to liability. These risks spanned the design, content, compatibility with existing health care services, promotion, and usage of mHealth technologies. Participants described liability as encompassing daily practice challenges, including patient deterioration due to delayed responses, unclear service scopes, and fragmented health care systems. These liability issues were described as extending beyond individual responsibilities, necessitating systematic or structural identification and resolution. Health care professionals across regions and institutions consistently highlighted liability as a potential barrier to the broader application and acceptance of mHealth if left unaddressed.

We have this ‘hip fracture app’ mostly introduced to patients by physical therapists after assessment. The exercise videos (in the app) start with disclaimer. But it won’t protect nurses if we recommend this app to patients and accident happens. “Even recommended by physiotherapists, what if patient has an accident while following the videos?

Participant from a community health center in Hong Kong SAR

Some patients from other provinces are treated at our hospital. When we provide follow-up contacts online, the advice we give may be incompatible with the practices in their province. This can lead to non-adherence and delays in treatment.

Nurse from a general tertiary hospital in Wuhan

#### Timeliness to Regulate Liability

Participants from Wuhan repeatedly emphasized timeliness in responding to health requests via mHealth and the liability risks associated with delayed responses, which emerged as a recurring subtheme within liability. They emphasized that mHealth often involves time-sensitive health requests from patients, yet delays can arise from issues such as internet connectivity or pending auxiliary examinations. Professionals from Wuhan voiced concerns over the ambiguity of response responsibilities and timeframes. Striking the right balance is crucial, as stringent timelines could extend professionals’ working hours, while leniency might jeopardize patient safety. They stated the need for clear guidelines, effective system designs, and institutional support to mitigate liability risks and ensure the safe integration of mHealth into health care provision.

We have a consultation app, but it's not certain that you will get a reply immediately after asking a question. If the response time exceeds around 24 hours, the system will close the request and return the consultation fee. Patients can initiate a new consultation request, but as time goes by, the problem may remain unresolved. What if deterioration happens during the waiting?

Nurse from a general tertiary hospital in Wuhan

### Theme 2: Change Management

Health care professionals emphasized that the safe integration of mHealth into real-world settings requires effective change management at both individual and institutional levels. Four subthemes emerged: appropriate referral, resources and funding, informed mHealth usage, and working process efficiency. While the first 3 were viewed as primarily benefiting patients, working process efficiency was regarded as critical for health care professionals. Comparative analysis revealed a notable absence of concerns regarding resources and funding among participants from Hong Kong SAR. In contrast, these participants notably focused more on informed mHealth usage and efficiency improvements compared to their counterparts in Wuhan.

#### Appropriate mHealth Referral

Participants stated that mHealth expands referral options, requiring professionals to adopt a patient-centered approach that considers cultural and personal preferences, individual capacity, willingness to use digital platforms during care provision, and digital divides and literacy when introducing mHealth. They highlighted the growing need to direct patients to trustworthy mHealth resources, given the abundance of online health information. Participants also provided examples of patient harm resulting from accessing misleading health information on mass media, highlighting the need for health care professionals to make appropriate mHealth referrals.

I remember one patient who underwent percutaneous coronary intervention watched ureteral stent videos online. He became furious and upset, questioning why we left the stent in his body. We need to refer them to the appropriate mHealth platform.

Nurse from a general tertiary hospital in Wuhan

Nowadays the usage of electronic devices is widespread, older people can generally accept you are using the phone or iPad to help them or even teach them to use it.

Nurse from a community health center in Hong Kong SAR

#### Improving Human Resources and Funding Allocation

Participants from Wuhan expressed their concerns on a lack of human resources and funding support, stressing the potential risks to patient safety and mHealth sustainability. They noted the cost-saving benefits of mHealth for patients, such as obtaining health education remotely and reducing the expenses associated with in-person health care visits and better health outcomes. However, they also elaborated that it might lower hospital revenue, creating a “conflict of interest” that can jeopardize the quality and sustainability of mHealth.

Looking back at the pandemic, some patients reduced their hospital visits by using mHealth to address health issues. This led to a reduction in the hospital's revenue, but from the patient's perspective, their benefits were certainly enhanced.

Physician from a general tertiary hospital in Wuhan

Other participants also echoed concerns about funding and resource limitations, describing how they are using their personal time and devices to provide free mHealth services. They emphasized the need to prioritize health care provision in face-to-face settings over mHealth encounters. Therefore, patients should consider mHealth as a supplementary tool to avoid potential harm.

When patients and I become friends on WeChat (a social media app), I make it clear that I am using my personal time for the consultation. We need dedicated devices for mHealth provision instead of using our own phones, as the mixture can cause confusion.

Nurse from a general tertiary hospital in Wuhan

#### Promoting Informed mHealth Usage

Participants shared that the use of mHealth and other technology platforms is an inevitable trend. They emphasized that professionals need to be trained and informed about the advantages, limitations, and associated risks of different platforms so that they can properly educate patients during implementation. They expressed that their current experiences are based on trial and error, but more proactive approaches are needed for real-world usage. In this way, both professionals and patients can exchange views and preferences to promote informed mHealth usage.

Some patients prefer e-wound consultations. But it only allows us to see pictures, not the wound swabs, debridement, odor, and skin texture, which require in-person visits. In this case, we need to inform them mHealth can delay treatment.

Nurse from a community health center in Hong Kong SAR

#### Enhancing Efficiency

Participants unanimously shared that mHealth platforms improved the teamwork process, making interprofessional communication more efficient and accurate, thereby reducing risks. This was highlighted by participants from Hong Kong SAR, who noted that granting them access to patients’ health records via mHealth made the work process more integrated and cooperative.

One of the advantages of using mHealth is that we can send messages/photos to our colleagues for instant feedback.

Physician from a general tertiary hospital in Wuhan

I highly encourage patients to use mHealth. Patients can see all their follow-up appointments and medication records. Our home visits have become much more efficient. If I teach a patient to use it, it can be inconvenient if the next community nurse visiting them does not know how to use it.

Nurse from a community health center in Hong Kong SAR

One participant also shared how mHealth helps to manage occupational exposure risk and prevent spreading infection.

mHealth will show alerts, for example, if a patient is VRE positive or has shingles. I can then alert the contact precautions and instruct caregivers to wear gloves before interacting. Also (notify the home health service team to) stop their home services until recovery.

Nurse from a community health center in Hong Kong SAR

### Theme 3: Ensuring Understanding

Participants highlighted that patient safety in mHealth depends not merely on providing information but on ensuring that patients truly understand it. They noted that while mHealth offers convenience and popularity, significant digital disparities, especially among older adults and underserved populations, pose serious risks. Participants stressed that overlooking these disparities could turn mHealth into a hazard by excluding vulnerable groups or providing generalized information that might mislead and harm patients.

We get used to mHealth everywhere—online booking, consultation, e-promotion. We may ignore that some people cannot even scan QR code to fill in basic information, or they do not even use smartphones. If we forget that, mHealth becomes a risk because we exclude deprived populations completely.

Nurse from a general tertiary hospital in Wuhan

The apps’ information is too general. For instance, it suggests patients with urinary catheters drink more water daily, 1.5 to 2 liters, and eat more fruit and vegetables. But for some people with renal failure who need fluid restrictions, or those with diabetes who can't eat too much fruit, this advice isn't suitable. There is some risk.

Nurse from a community health center in Hong Kong SAR

Without ensuring understanding, participants expressed that mHealth places professionals in challenging positions, leaving patients to independently interpret and adopt health behaviors, often with insufficient support. To address these challenges, participants advocated for face-to-face guidance before introducing mHealth tools.

There are several operational steps for tele-consultation, some older adults cannot figure out, so they call us for help. But they have hearing decline, we have to yell at the phone repeating same sentences. And they couldn’t understand. It’s frustrating.

Nurse from a general tertiary hospital in Wuhan

We will first teach the patient or caregiver how to use it face-to-face. Then, the QR code is provided for them to review and refresh their memory. If you only give the patient or caregiver a QR code to learn from, they could make mistakes. In such cases, they might claim they were just following the video instructions.

Nurse from a community health center in Hong Kong SAR

Few participants shared that they tend to avoid mentioning mHealth to patients who have difficulty understanding the technology or educational content in order to avoid risks.

It can be difficult for elderly to accurately input data because there are many steps, and the phone screen is small. Also, teaching them how to use it can be time-consuming. I won’t mention it unless the patient's caregiver is very young and familiar with apps, and has already downloaded HA Go.

Nurse from a community health center in Hong Kong SAR

The need for proper training was especially pronounced when using wearable devices to remotely track health data. Participants emphasized empowering patients or their caregivers to correctly use these devices, including providing hands-on training and asking for return demonstrations.

I believe the most important thing about using these devices is empowering patients or their caregivers to use them. We teach and ask for a return demonstration. We are not always beside them to guide or help. For instance, if they take vital signs wrongly, it could affect the readings and interpretation.

Nurse from a community health center in Hong Kong SAR

### Theme 4: Ensuring Information Security

Participants consistently highlighted the importance of information security, emphasizing that health information is sensitive in nature and that information leakage could bring both ethical and economical risks for patients. They shared that mHealth usage involves a health communication platform, which includes sociodemographic and clinical backgrounds, and diagnostic and treatment suggestions. Participants identified different scenarios where information leakage might occur, including device loss by professionals, patients inadvertently sharing information while seeking technical support, and oversharing through social media apps. Notably, differences emerged between the perspectives of professionals from Hong Kong SAR and those from Wuhan. Participants from Hong Kong SAR emphasized existing regulatory policies designed to protect patient information. They described strict measures for accessing medical systems and protocols for handling devices containing sensitive data. In contrast, professionals from Wuhan expressed the need for stricter oversight and governance of information security, particularly given the widespread use of social media platforms like WeChat and apps developed by commercial entities. They advocated for regulatory authorities, such as the public security bureau, to monitor and record activities to mitigate risks. Despite the differences, participant narration delineates the need for standard practice guidelines or standards across regions to ensure secure use of mHealth, which is tailored to local contexts and technological infrastructure. Participants from both regions recognized the potential risks of using mHealth platforms that store and communicate sociodemographic, clinical, and treatment-related data. Participants noted the shared responsibility of health care providers, regulatory bodies, and developers in ensuring robust data protection mechanisms.

Our hospital has professional-led patient group chat on WeChat, the response speed is better than other hospitals using apps in Wuhan. The risk management is necessary. I think relevant departments, especially the public security bureau, should at least have a record of it because you don't know how the group might develop.

Physician from a general tertiary hospital in Wuhan

Despite its hard to access the medical system without login credentials. We all need to take good care of our devices, such as phones and iPads. If lost, it is necessary to report it to the police. The department has regulations stating that patient personal identifiers cannot be taken outside. iPads must be placed in a zippered bag, and the bag must be attached to the nursing bag with a strap.

Nurse from a community health center in Hong Kong SAR

## Discussion

### Principal Findings

The integration of mHealth into health care systems has rapidly gained momentum as a global trend, offering promising opportunities to enhance both patient care and operational efficiency. As health care systems increasingly adopt mHealth solutions, it becomes crucial to understand the implications for patient safety in these new care models. This qualitative study is among the first to explore health care professionals’ perspectives on the safety concerns associated with mHealth implementation in real-world settings. The findings emphasize the importance of patient safety in ensuring that mHealth is integrated into a reliable and sustainable care model. Across participants from Hong Kong SAR and Wuhan, common themes emerged regarding safety challenges and the strategies for managing these concerns. However, regional differences also became apparent, highlighting the need for context-specific adaptations to effectively address local health care needs and conditions. These findings not only contribute to the growing body of knowledge on mHealth but also provide valuable insights for policy makers and health care providers as they navigate the complex process of integrating mHealth into diverse health care environments.

Findings from this study highlighted liability considerations pertaining to mHealth across settings, which differ from the liability statements of traditional health care settings that detail professional malpractice and compensation to patients [44]. Health care professionals’ discussions on liability were not intended to identify a responsible individual when risks occur, which could drastically hinder mHealth usage, but rather to acknowledge the inherent limitations and advocate for caution when adopting mHealth. Unlike a previous study on real-world mHealth usage where professionals and managers expressed concerns about the trustworthiness of apps [45], findings from this study highlighted the intrinsic limitations of mHealth in ensuring accurate health information exchange to enable prompt patient evaluation and decision-making. This difference may be attributed to the fact that participants from Hong Kong SAR used mHealth apps developed, operated, managed, and financed by the Hospital Authority, while participants from Wuhan were regarded as high-caliber professionals from top hospitals who translated their expertise in delivering in-person care to mHealth. Moreover, owing to the self-finance attributes of mHealth care provision in Wuhan [46], participants emphasized the importance of explicitly communicating the estimated timeframe and responding in a timely manner to enhance reliability.

The study also revealed the vital role of change management in implementing mHealth in real-world settings. Consistent with existing literature [47,48], participants unanimously recognized mHealth as an inevitable trend due to its potential to enhance patient outcomes and professional efficiency. Growing acceptance and usage of technology among patients further motivated professionals to recommend appropriate mHealth platforms [47]. Likewise, health care professionals should be trained and informed about existing reliable mHealth options and be prepared to educate patients about the potential benefits and risks of using them [49]. Effective change management, as suggested by participants, should account for variations in the readiness of professionals to adopt mHealth, emphasizing the importance of training and education to inform both professionals and patients about the benefits and limitations of mHealth. Even health care professionals who hesitate to use mHealth should be empowered to learn and prepare themselves to partner with colleagues and patients who desire using mHealth for health purposes to ensure care consistency and patient safety.

Findings from this study affirmed the literature’s emphasis that mHealth care provision should ensure the understanding of patients to empower engagement and generate health benefits [50,51]. Unlike interventional studies that consider multiple factors to manage confounding variables, participants in this study consistently suggested mobilizing various resources, such as volunteers for technology coaching, informal caregivers, and in-person teachers for return demonstrations, to improve comprehension and prevent risks in real-world mHealth implementation. Theoretically, mHealth literacy should be evaluated and promoted to ensure equity in mHealth implementation, while in practice, health care professionals’ evaluations of patients’ mHealth literacy could determine whether they use or recommend mHealth. Consistent with the literature, this study mentioned the usage challenges faced by patients, such as small font sizes for older adults, small touch screens to operate, and learning difficulties [14]. The need for professionals to repeatedly teach patients to operate technology and address potential misunderstandings could dampen the confidence of professionals and compromise their willingness to use mHealth for care provision.

Information security, though less prominently discussed, emerged as a high priority to meet legislative requirements, which is consistent with a previous study that revealed the perspectives of stakeholders in real-world mHealth service provision [52]. This aspect was considered particularly important when participants mentioned the tendency of linking health records from health care settings with mHealth platforms, the collection of telemonitoring data, and the exchange of multimedia information between professionals. However, contrary to a previous study where managers considered data security to be outside their direct role [52], participants in this study shared experiences of themselves proactively implementing strategies to prevent information leakage.

### Implications

The findings from this study could enhance the understanding of the safety aspects associated with real-world mHealth service provision and inform essential change management at the individual, process, and institutional or policy levels. Despite collecting data regionally, the study findings could have international applicability as patient safety is holistic and systematic, highlighting the universality of human perspectives and strategies to enhance safety through the establishment, interactions, experiences, and outcomes of mHealth. Patient safety remains the first and foremost priority in mHealth care provision, necessitating effective communication, care coordination, and multidisciplinary collaboration to ensure care continuity [53]. The results could potentially inform developers and user interaction designers to include more safety-enhancing features for real-world mHealth implementation, especially to make it user-friendly and inclusive for people with pre-existing ailments or older adults. Despite variations in mHealth apps and their integration into health care systems, the professional skills and user views identified in this study could hold significant potential for advancing international health care. This study also has policy implications. Health policy makers in Hong Kong SAR may consider redefining the liability of mHealth and cautioning against the inherent limitations of this model to promote informed usage and appropriate mHealth referral. Health policy makers in mainland China may consider expanding guiding policies to include not only diagnosis and treatment but also patient consultation, self-care empowerment, and related data security measures. The settings may support the training of health care professionals to cultivate smart manpower and streamline care processes to provide safe mHealth services.

### Limitations

While this study offers valuable insights into the patient safety aspects of mHealth in real-world health care settings, it is not without limitations. First, the study’s qualitative design limits the generalizability of the findings, as the data were collected from health care professionals in 2 specific regions, Wuhan and Hong Kong SAR. The findings may not fully reflect the experiences or perspectives of health care providers in other regions with differing health care systems or technological infrastructure. Moreover, excluding health care professionals in nondirect care roles may omit important perspectives. Future studies should consider involving leaders, health informatics personnel, and other related parties to capture broader views. While the multicenter approach enhanced the breadth of perspectives, the findings might not capture the full diversity of health care professional experiences across different health care settings, particularly in smaller or rural environments. While the study focused on health care professionals’ perspectives, it did not include the views of students, support staff, leaders or managers, patients, or caregivers, who play critical roles in mHealth adoption and usage. Future research should include a broader range of stakeholders. Lastly, the samples from the 2 health care systems were not exactly homogeneous, as we recruited both physicians and nurses from Wuhan and only nurses from Hong Kong SAR (despite several rounds of attempts to recruit physicians), which may challenge the stability of the results. This difference arose because mHealth platforms are co-created and co-operated by physicians and nurses from the same departments in Wuhan, whereas the division of care provision in Hong Kong SAR is clear.

### Conclusion

The widespread adoption of mHealth technologies is reshaping health care delivery, bringing both significant opportunities and challenges. Health care professionals recognized the importance of patient safety in mHealth usage and described key considerations for ensuring patient safety. They recommended different competencies and shared commendable practices and policies for delivering mHealth care safely. Institutional or system-level support is needed to clarify the scope and liability of mHealth to facilitate informed usage, improve change management, and ensure information security. Furthermore, it is crucial for health care professionals to foster greater technology acceptance, make informed referrals, and strengthen their competencies in ensuring patient comprehension in order to maintain the safety and sustainability of mHealth care.

## Appendix

Multimedia Appendix 1 Interview guide.

## Abbreviations

mHealth: mobile health
SAR: Special Administrative Region

## Data Availability

The data analyzed in this study are available from the corresponding author upon reasonable request.
