# Supplementary material for: Real-World Mobile Health Implementation and Patient Safety: Multicenter Qualitative Study
Source: J Med Internet Res. 2025 Apr 29;27:e71086. doi: 10.2196/71086 (PMC12076031; doi:10.2196/71086)
Supplement: Multimedia Appendix 1 [file jmir_v27i1e71086_app1.docx]

**Multimedia Appendix 1.** Interview guide.

1. What’s your experiences in using the mHealth (e.g., mobile apps, social media, wearable devices) in providing healthcare services?
2. What are the safety aspects you have encountered or observed when using these mHealth platforms? (Probes: stories on training towards its usage and problem-solving/ interacting with users or colleagues/ data sharing and storage/ compatibility with existing service/ tele-monitoring and tele-coaching related issues, reliability of automatic chatbot)?
3. How would you handle those safety aspects (Probes: What are the existing strategies/ what are the available resources and support/ what are the follow up evaluation and measures/ how do you feel about it?)
4. What are the enablers for providing mHealth safely?
5. What are the barriers to providing safe mHealth care?
6. What’s your suggestions towards improving the safety concerns (probes: at the institutional/governing level, at the process of implementation, at the individual level)?
